# Strong Intermixing Effects of LFO_1−x_/STO_x_ toward the Development of Efficient Photoanodes for Photoelectrocatalytic Applications

**DOI:** 10.3390/nano13212863

**Published:** 2023-10-29

**Authors:** Yassine Nassereddine, Manal Benyoussef, Nitul S. Rajput, Sébastien Saitzek, Mimoun El Marssi, Mustapha Jouiad

**Affiliations:** 1 Laboratory of Physics of Condensed Matter, University of Picardie Jules Verne, Scientific Pole, 33 Rue Saint-Leu, CEDEX 1, 80039 Amiens, France; yassine.nassereddine@u-picardie.fr (Y.N.);; 2 Advanced Materials Research Center, Technology Innovation Institute, Abu Dhabi P.O. Box 9639, United Arab Emirates; 3 Catalyse et Chimie du Solide (UCCS), University of Artois, CNRS, Centrale Lille, ENSCL, UMR 8181, 62300 Lens, France

**Keywords:** LaFeO_3_, SrTiO_3_, photoelectrochemical measurements, water splitting, reversible hydrogen electrode (RHE), photoanode

## Abstract

Aiming to improve the photocatalytic properties of transition metal perovskites to be used as robust photoanodes, [LaFeO_3_]_1−x_/[SrTiO_3_]_x_ nanocomposites (LFO_1−x_/STO_x_) are considered. This hybrid structure combines good semiconducting properties and an interesting intrinsic remanent polarization. All the studied samples were fabricated using a solid-state method followed by high-energy ball milling, and they were subsequently deposited by spray coating. The synthesized compounds were demonstrated to possess orthorhombic (Pnma) and cubic (Pm$\bar{3}$m) structures for LFO and STO, respectively, with an average grain size of 55–70 nm. The LFO_1−x_/STO_x_ nanocomposites appeared to exhibit high visible light absorption, corresponding to band gaps of 2.17–3.21 eV. Our findings show that LFO_0.5_/STO_0.5_ is the optimized heterostructure; it achieved a high photocurrent density of 11 μA/cm^2^ at 1.23 V bias vs. RHE and an applied bias photo-to-current efficiency of 4.1 × 10^−3^% at 0.76 V vs. RHE, as demonstrated by the photoelectrochemical measurements. These results underline the role of the two phases intermixing LFO and STO at the appropriate content to yield a high-performing photoanode ascribed to efficient charge separation and transfer. This suggests that LFO_0.5_/STO_0.5_ could be a potential candidate for the development of efficient photoanodes for hydrogen generation via photoelectrocatalytic water splitting.

## 1. Introduction

Worldwide energy consumption has significantly increased due to rapid technological advancements, accelerating industrialization, and economic expansion [1,2,3,4]. Today, fossil fuels remain the world’s most used energy source [5]. However, it is inconceivable to disregard the damage these fuels are causing to the environment and human well-being by releasing toxic pollutants [6,7]. Hence, prioritizing the development of alternative clean energy supplies becomes crucial to cope with the increasing global energy demand while facilitating the transition towards sustainable and environmentally friendly energy use [8]. In this context, the deployment of hydrogen (H_2_) fuel is regarded as a suitable alternative solution to produce clean energy from green resources, such as water and solar, by means of photocatalysis water splitting (WS) [9,10,11,12,13]. Furthermore, using solar power to generate green H_2_ presents a promising approach for renewable energy production. In particular, photoelectrochemical (PEC) WS, which utilizes sunlight to produce H_2_, has attracted great attention in the last few years because of its potential to achieve high solar energy to H_2_ conversion (STH) while operating at low temperatures [14,15]. However, in order to obtain an effective PEC WS process, it is necessary to develop a high-performing photocatalyst, which exhibits the ability to efficiently generate charge carriers and electron–hole (e-h) pairs to enable high WS yield by enhancing the hydrogen evolution (HER) and oxygen evolution (OER) reactions.

In this regard, several photocatalysts have been thoroughly studied and evaluated for high WS reaction yield. For instance, metal oxide photocatalysts, e.g., TiO_2_, ZnO, and Fe_2_O_3_, among others, have been widely probed for PEC WS owing to their stability, good yield, and, most importantly, low cost [16,17,18,19,20,21,22]. In contrast, their use as efficient photocatalysts is still limited by their ability to absorb light in the UV region (4% of total sunlight), which significantly reduces their STH efficiency. Several strategies have been adopted to overcome these limitations by enhancing their capabilities, such as the development of heterojunction-based photocatalysts, or their combination with plasmonic materials, in order to extend their operation in the full sunlight spectrum, hence augmenting the WS reaction yield [23,24,25,26,27,28,29].

Recently, oxide perovskite materials (PMs) have received and sparked great attention due to their distinctive electrochemical and photophysical properties [30,31,32,33] owing to their dual properties, such as having an appropriate band gap for WS and intrinsic polarization to drive the WS reaction. For instance, a SrTiO_3_ (STO) n-type semiconductor having a wide band gap of ~3.2 eV has been widely investigated for H_2_ production by solar-driven WS [34,35]. Due to the STO’s large band gap, the rapid electron–hole pair recombination, and the active back-reactions, STO has shown some weaknesses regarding STH efficiency. Alternatively, doped STO or its use in conjunction with a cocatalyst were reported to be interesting routes to enable the STO band’s alignment edge with the water redox potentials, leading to increased STH efficiency [36,37,38]. In particular, using a solid-state synthesis process, BFO-xSTO (x > 0.1) compounds yielded high optical absorption (>80%) and a photocurrent density of ∼0.17 μA cm^−2^ obtained for x = 0.15 at 0 bias [39].

Lately, other PM semiconductors exhibiting narrow band gaps in the 2.0–2.6 eV range have attracted attention in the photodegradation of organic pollutants. For instance, LaFeO_3_ (LFO) has showcased its ability to photodegrade organic dyes and split water molecules when exposed to visible light illumination [40,41]. Yet, the effectiveness of single-phase LFO material for photocatalysis is also limited by the short hole diffusion length, L_p_, which is determined by the redox and electrical behavior of the B-site cation (Fe^3+^) [42]. To remedy this issue, LFO has demonstrated a high ability to delay the charge recombination when it is coupled with a wide band gap semiconductor [43]. These findings have opened the door for further development strategies to improve the photocatalytic properties of LFO.

In this context, the present work reports on the elaboration of LFO_1−x_/STO_x_ compounds, x = 0, 0.25, 0.5, and 1, by solid-state-assisted high-energy ball milling to identify the most suitable compound for high WS yield. In this sense, the fabricated compounds were subsequently deposited by spray coating and evaluated for photocatalytic WS reactions.

## 2. Materials and Methods

**Figure 1:**
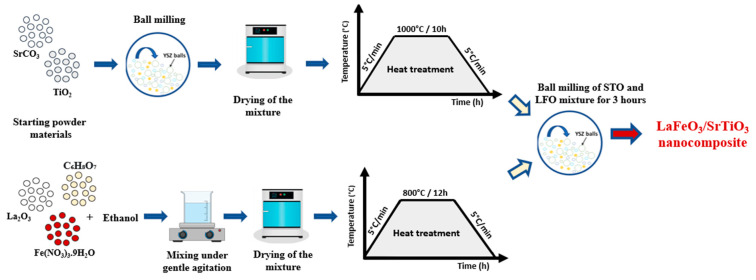
Steps of LFO_1−x_/STO_X_ nanocomposite preparation.

The LFO compound was prepared by a solid-state method using 0.67 g of lanthanum (III) oxide, 99.9%, 1.66 g of iron (III) nitrate nonahydrate, >98%, and 2.3 g of citric acid, 99+%, from ThermoFisher Scientific. The mixture was diluted in ethanol under gentle magnetic stirring (500 tr/min) for 2 h to obtain a homogeneous solution. The resulting solution was then heated to 100 °C and maintained for 5 h. The LFO foam was ground in an agate mortar before annealing in the air for 12 h at 800 °C. The STO compound was prepared using a stoichiometric amount of strontium carbonate, SrCO_3_ > 99.9%, and titanium dioxide, TiO_2_ > 99%, from Sigma Aldrich, mixed in ethanol and further ball milled at a high energy of ~1000 rpm for 2 h (Retsch GmbH, Haan, Germany). The dried powder was subsequently ground in an agate mortar and calcinated in the air for 10 h at 1000 °C at a heating rate of 5 °C/min. Several steps were necessary to elaborate the LFO_1−x_/STO_x_ nanocomposite, as illustrated in Figure 1.

All the samples were prepared by mixing appropriate contents of LFO and STO in ethanol, followed by high-energy ball milling operating at 1000 rpm for 3 h to homogenize the mixture and further reduce the particle size. 

To examine the optical, electrical, and photocatalytic properties of the synthesized compounds, a spray-coating technique was employed to deposit the nanoparticles made of these compounds onto a conductive and transparent substrate made of fluorine-doped tin oxide (FTO). For all the considered samples, a precursor solution was prepared with a molar concentration of 0.1 M in ethanol, which was carefully diluted by stirring the solution in an ultrasonic bath for 1 h. During the deposition process performed at a 1 mL/min rate, the FTO substrate was maintained at a temperature of 400 °C.

The structural characterization and the phase purity of all the compounds were examined using an X-ray diffractometer (D4 Endeavor, Bruker) equipped with a 0.15 nm CuKα source. The analysis of the samples’ vibrational modes was carried out using a micro-Raman spectrometer (Renishaw) under green laser excitation at 532 nm. The materials’ microstructure and morphology were examined by environmental scanning electron microscopy (ESEM), Quanta 200 from ThermoFisher Scientific, and the elemental composition was analyzed using energy-dispersive X-ray spectroscopy (EDS) (Oxford Instruments). To assess the crystal structure, the nanoparticle size, and the lattice parameters, high-resolution transmission electron microscopy (HRTEM) analysis was performed on all the samples using an image Cs-corrected TEM system, Titan G2 (ThermoFisher Scientific), operating at 300 kV. The optical properties of the samples were determined using a UV–Vis-near IR spectrometer (JASCO V-670) in the spectral range of 300–1000 nm. Finally, the photoelectrochemical (PEC) measurements and electrochemical impedance spectroscopy (EIS) were conducted on PalmSens4 (Houten, The Netherlands) using a cell consisting of a three-electrode working electrode (WE), where our elaborated materials are placed, a Ag/AgCl reference electrode (RE), and a Pt fishnet as a counter electrode (CE). The electrolyte was an aqueous solution of tap water. 

## 3. Results and Discussion

### 3.1. Structural and Vibrational Analyses 

**Figure 2:**
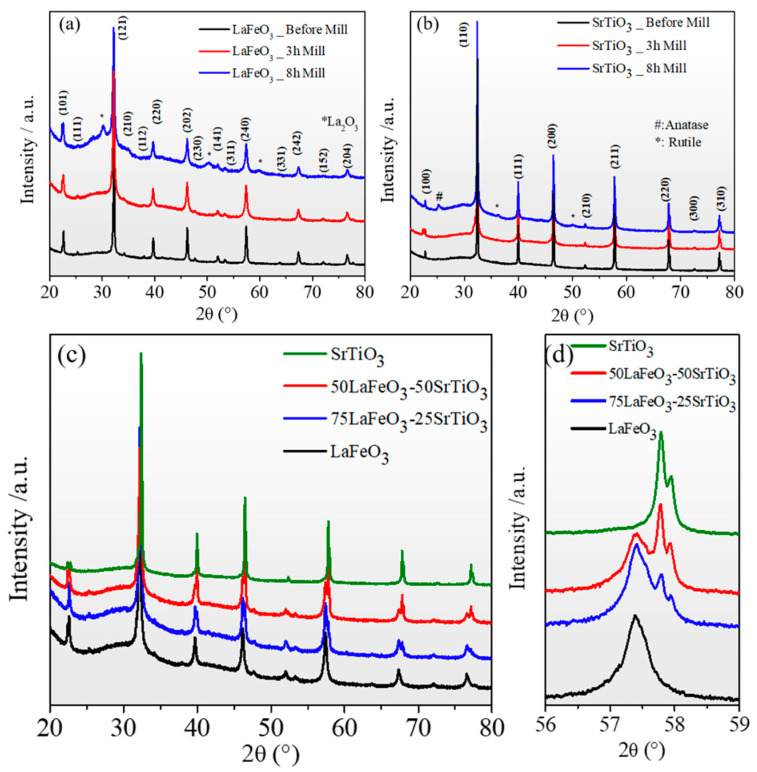
XRD patterns of (**a**) LaFeO_3_ for different ball milling times; (**b**) SrTiO_3_ for different ball milling times; (**c**) LFO_1−x_/STO_x_ nanocomposites after 3 h ball milling; (**d**) zoomed-in XRD patterns centered around 57.5°.

The XRD diagrams obtained for the SrTiO_3_ and LaFeO_3_ powders, as fabricated and after 3 h and 8 h of ball milling, recorded in the range of 20–80° and are shown in Figure 2a,b. All the peaks are indexed by referring their positions to the JCPDS crystal structure database (No. 073-0661 for SrTiO_3_ and No. 37-1493 for LaFeO_3_) [44,45]. Before and after 3 h milling, high-purity perovskite phase patterns were observed for both samples. The LFO exhibits an orthorhombic crystal structure with cell dimensions of a ≈ 5.563 Å, b ≈ 7.8430 Å, and c ≈ 5.552 Å, consistent with the space group (Pnma) (62), and the typical reflections for the STO phase were present at (100), (110), (111), (200), (210), (211), (220), and (310), which indicates the presence of the cubic (Pm$\bar{3}$m) symmetry (221) with a lattice parameter, a ≈ 3.90 Å. In contrast, the samples milled for 8 h showed the presence of secondary phases, such as La_2_O_3_ for the LFO phase and TiO_2_ anatase and rutile for the STO. 

Figure 2c depicts the room temperature X-ray diagrams of the LFO_1−x_/STO_x_ nanocomposite samples milled for 3 h. Both the LFO and STO phases were obtained for x = 0.25 and x = 0.50. The zoom-in of the peak, centered at 57° of the LFO_1−x_/STO_x_, shows a broadening peak for both LFO_0.5_/STO_0.5_ and LFO_0.75_/STO_0.25_, which indicates a reduced crystallite size (Figure 2d). 

**Figure 3:**
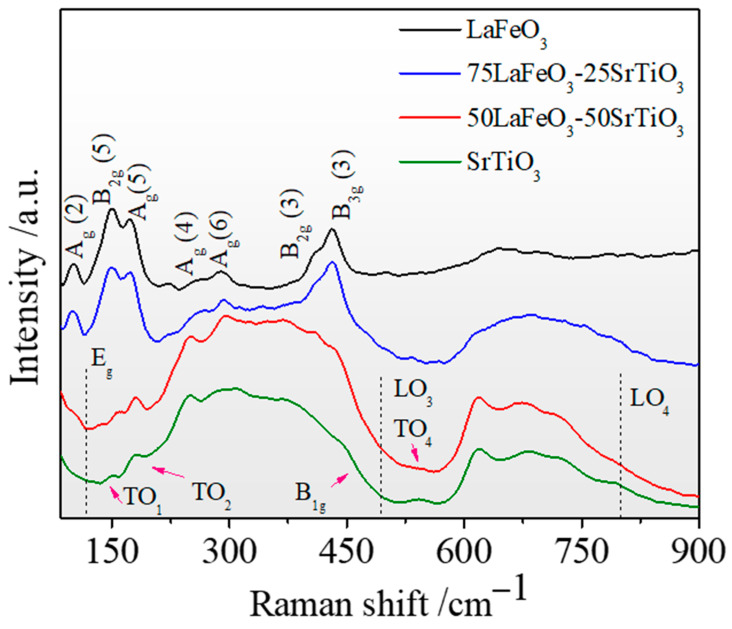
The Raman spectra of LFO_1−x_/STO_x_ nanocomposites collected at room temperature.

The Raman spectra of the synthesized LFO_1−x_/STO_x_ powders are depicted in Figure 3. The low-energy bands for all the samples are shown, tagged according to the A_g_ and B_2g_ symmetry of the Pnma space group for the LFO, and the corresponding E_g_ and B_1g_ modes observed in the STO with the space group Pm$\bar{3}$m. 

Typically, the modes induced by La vibration tend to rise at frequencies lower than 200 cm^−1^. In addition, in the 200–300 cm^−1^ range, the modes are attributed to the oxygen octahedral tilt in the La. Alternatively, the modes that fall between 400 and 450 cm^−1^ correspond to the oxygen octahedral bending vibrations, while those exceeding 500 cm^−1^ pertain to the oxygen stretching vibrations. At ambient temperatures, the cubic symmetry of STO precludes the occurrence of first-order Raman scattering. A weak mode is also observed at 478 cm^−1^, attributed to the LO_3_ vibration mode. Furthermore, it is worth noting that two additional modes are observed at 125 cm^−1^ and 450 cm^−1^, matching with the E_g_ and B_1g_ modes observed in the pure STO. The remaining Raman vibrational modes are subsequently identified at 175, 545, and 790 cm^−1^ as TO_2_, TO_4,_ and LO_4_, respectively.

**Figure 4:**
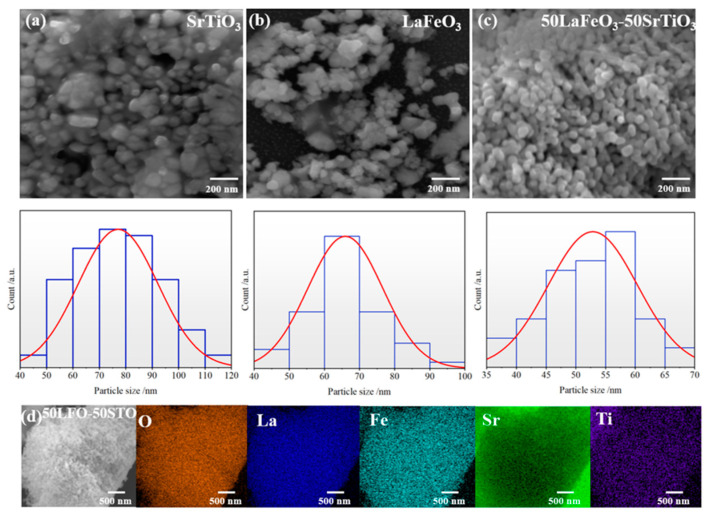
SEM images of sample powders and the corresponding particle size distribution for (**a**) STO, (**b**) LFO, and (**c**) LFO_0.5_/STO_0.5_ and its corresponding (**d**) EDS map.

To examine the samples’ microstructure, the specimen powders were dispersed in ethanol; then, a few solution drops were subsequently deposited on the flat silicon substrate. Figure 4 depicts the SEM images of the samples of LFO_1−x_/STO_x_, which were initially ball milled for 3 h. The grains have a spherical shape with a Gaussian distribution in their size. The grains of STO and LFO appear to have an average size of 70 nm and 65 nm, respectively, compared to 55 nm for the LFO_0.5_/STO_0.5_ sample. 

The nanocomposite sample of LFO_0.5_/STO_0.5_ was selected for further examination by EDS to highlight the co-existence of both STO and LFO using La, Fe, Sr, and Ti as markers. As can be seen in the EDS map, the elements’ contents and the typical EDS spectrum are given in Figure S2 in the Supplementary Information. The base elements of the nanocomposite were recorded as shown in the EDS maps of Figure 4d.

**Figure 5:**
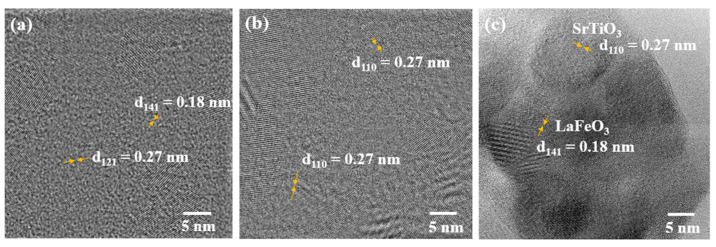
HRTEM images of LFO_1−x_/STO_x_ nanocomposites: (**a**) LaFeO_3_, (**b**) SrTiO_3_, (**c**) LFO_0.5_/STO_0.5_.

To further assess the crystal structure of the samples, HRTEM analysis was carried and typical images for pristine STO and LFO as well as for the nanocomposite LFO_0.5_/STO_0.5_ are given in Figure 5. 

The orientation of the grains can be used to estimate their size (10 to 30 nm range) for the samples, smaller than those determined using the SEM images. The d spacings of 0.27 nm and 0.18 nm correspond to the pristine LFO (121) and (141) planes, respectively, and 0.27 nm corresponds to the STO (110) plane. The analysis of the interconnected LFO and STO particle regions in the LFO_0.5_/STO_0.5_ nanocomposite (Figure 5c) revealed the d-spacings of both the LFO (0.27 nm) and the STO (0.18 nm). These findings are in accordance with the XRD diffraction and Raman results. We expect that the LFO/STO interface heterojunction would promote an efficient charge transfer.

### 3.2. Optical Properties 

**Figure 6:**
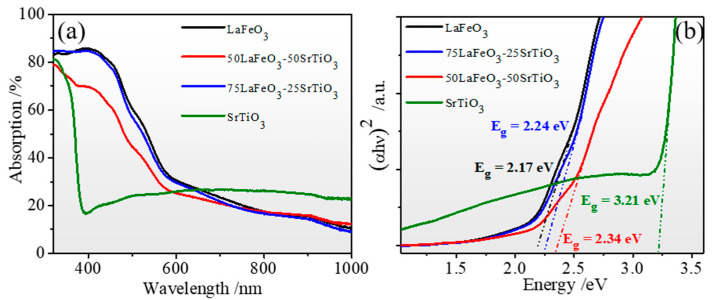
Optical properties of LFO_1−x_/STO_x_ films: (**a**) absorption and (**b**) Tauc plots of LFO_1−x_/STO_x_ films.

Figure 6 depicts the optical absorption for the pristine LFO, STO, and LFO_1−x_/STO_x_ nanocomposite samples deposited on the FTO substrate. The absorption was extracted from the optical transmittance and reflectance spectra provided in the Supplementary Information. The STO and LFO systems exhibited high transmittance of >60% in the visible- to near-infrared regions, and a maximum reflectance was recorded at 400 nm and 580 nm for the STO and LFO, respectively (Figure S1). As shown in the optical absorption plots of Figure 6a, the pristine LFO exhibited a high absorption (>80%) in the UV-blue region (300–450 nm), followed by a sharp decrease of 60%, up to 650 nm where a near-plateau behavior was observed up to the near-infrared domain. Surprisingly, the addition of 25% STO in the STO/LFO nanocomposite did appear to change its overall absorption in the full region of 350–1000 nm. However, the optical absorption of 50LFO-50STO nanocomposite revealed a decrease of 10 to 15% in the visible region, and equivalent light absorption to other compounds above 650 nm. On the other hand, the pristine STO exhibited lower optical absorption in the entire 380–600 nm region, where it attained its minimum (<20%) at 400 nm. Above 650 nm, STO appeared to exhibit slightly higher absorption compared to all the LFO-based nanocomposite samples. This clearly indicates that the intermixing between LFO and STO has a beneficial effect in increasing the overall light absorption and, in particular, in the visible region. The band gap of the samples was determined using the Tauc plot method (Figure 6b).

Similar to the reported band gap values [46], we obtained 3.21 eV for the pristine STO thin film. Then, the band gap was gradually reduced, with increasing amounts of LFO, to reach 2.34 eV, 2.24 eV, and 2.17 eV for the LFO_0.5_/STO_0.5_, LFO_0.75_/STO_2.5_, and neat LFO, respectively. 

### 3.3. Photoelectrochemical Measurements 

The photoelectrochemical measurements were carried out on the LFO_1−x_/STO_x_ films by determining the transient photocurrent using three electrodes under a solar simulator. The electrodes consisted of Ag/AgCl as the reference electrode, Pt fishnet as the counter electrode, and LFO_1−x_/STO_x_ films deposited on a fluoride tin oxide (FTO) substrate serving as the working electrode. An electrolyte solution of tap water was employed. During the light excitation, linear sweep voltammetry (LSV) and chronoamperometry experiments were conducted. The Nernst equation, given below, was used to evaluate the potential versus reversible hydrogen electrode (RHE):

E*_RHE_* = E*_Ag/AgCl_* + (0.06 × pH) + E_0_

E_0_ ≃ 0.197 V at 25 °C for pH = 7

**Figure 7:**
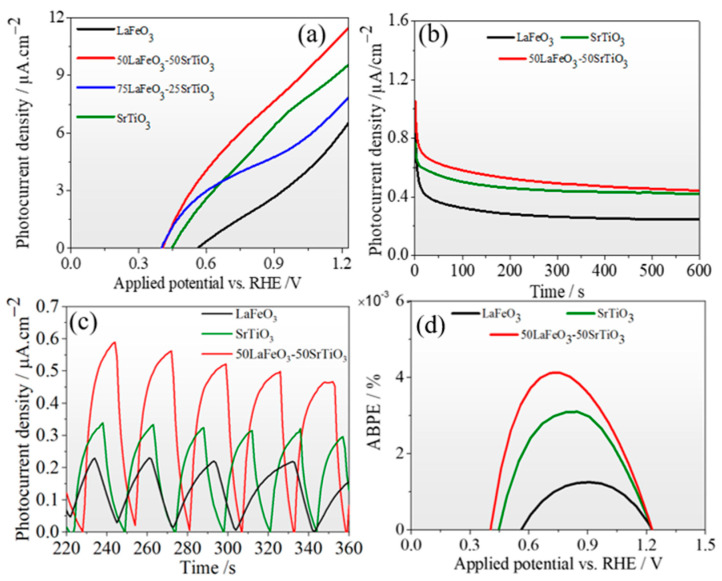
Photoelectrochemical measurements. (**a**) Photocurrent density versus potential (J–V) curves. (**b**) Steady state at 0.6 V versus RHE under halogen lamp excitation (100 mW/cm^2^). (**c**) Transient photocurrent responses. (**d**) Applied bias potential efficiency (ABPE) as a function of applied voltage versus RHE.

The LSV scanning rate was set to 0.1 V/s within a range of 0 to 1.23 V vs. RHE, representing the required theoretical value of the water redox potential. Figure 7a shows the photocurrent density as a function of potential versus RHE for all the samples; the highest photocurrent density was obtained for the LFO_0.5_/STO_0.5_, which continues to increase with increasing potential versus RHE to reach up to 11 μA/cm^2^ at 1.23 V vs. RHE. The onset potential of 0.56 V for the LFO_0.5_/STO_0.5_ and the observed shift toward more cathodic onset potentials suggest an improvement in charge transport, leading to higher separation efficiency, even at lower applied potential values. In addition, steady-state measurements of the generated photocurrent density, presented in Figure 7b, were used to assess the photocurrent density’s long-term stability using a bias potential of 0.6 V under halogen lamp illumination (100 mW/cm^2^). It was observed that a rapid drop occurred during the first 50 s, followed by a plateau, showing that the photocurrent had reached stable values of 0.58, 0.5, and 0.32 µA/cm^2^ for the LFO_0.5_/STO_0.5_, STO, and LFO. 

Figure 7c shows the transient photocurrent response of the LFO_0.5_/STO_0.5_, STO, and LFO recorded at a 0.6 V applied bias with the light on/off. As can be seen, there is an important photocurrent density achieved by the LFO_0.5_/STO_0.5_ (0.58 µA/cm^2^) nanocomposite compared to the bulk counterparts, STO (0.34 µA/cm^2^) and LFO (0.23 µA/cm^2^). This result demonstrates clearly the size effect in improving the charge separation. Furthermore, once LFO is associated with STO to create a heterojunction interface, we observe an interfacial charge separation enhancement, resulting in considerable improvement in the photocurrent density obtained for the LFO_0.5_/STO_0.5_ sample, attaining 0.58 μA/cm^2^. It is well known that there is a direct correlation between the photocurrent response and the separation efficiency of photogenerated electron–hole (e-h) pairs, meaning that higher photocurrent responses typically indicate higher e-h separation rates [47]. Figure 7d displays the applied bias photo-to-current efficiency (ABPE) of all the assessed photoanodes. The ABPE was determined using the J–V curve, as per this equation [48,49]:

$$ABPE=\frac{J_{ph}\times (1.23-V_{b\;})}{P_{total}}$$

where $J_{ph}$ is the photocurrent density, $V_{b\;}$ is the applied potential, and $P_{total}$ is the incident illumination power density. The ABPE obtained for the LFO_0.5_/STO_0.5_ was 4.1 × 10^−3^% at 0.76 V vs. RHE, which is much higher than those obtained for the STO (3.1 × 10^−3^% at 0.85 V) and the LFO (1.24 × 10^−3^% at 0.9 V). 

**Figure 8:**
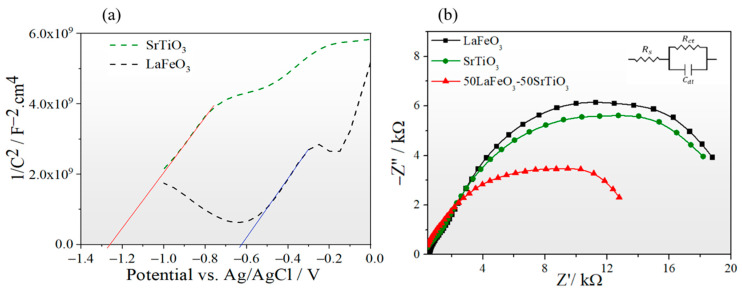
(**a**) Mott–Schottky plots of the SrTiO_3_ and LaFeO_3_ electrodes: Vfb = −0.62 V (blue line) and −1.23 V (red line) for LFO and STO, respectively (**b**) Electrochemical impedance measurements (EIS) of LFO_1−x_/STO_x_ samples (the inset shows the used Randles circuit).

In the following, the e-h separations, charge carrier mobility, and transfer are probed using Mott–Schottky and impedance spectroscopy (EIS) in an aqueous solution of Na_2_SO_4_ (0.1 M) and tap water, respectively. Figure 8a depicts Mott–Schottky plots of pristine STO and LFO, along with their respective electrochemical impedances recorded at 1 kHz. Both the STO and LFO exhibited positive slopes within the relevant voltage range considered for our photoelectrochemical measurements. This indicates that both samples are n-type semiconductors. Moreover, using the Mott–Schottky equation, the flat band potential and the charge carrier density were determined based on the M-S curves:

$$\frac{1}{C^{2}}=\frac{2}{\varepsilon \cdot \varepsilon_{0}{\cdot A}^{2}\cdot e\cdot N_{D}}(V-V_{fb}-\frac{k_{b}T}{e})$$

where C is the space charge layer capacitance, ε is the dielectric constant, ε_0_ is the permittivity of free space (8.854 × 10^−12^ F m^−1^), A is the electrode surface area, e is the electronic charge (1.602 × 10^−19^ C), N_D_ is the doping density, V is the applied potential, V_fb_ is the flat band potential, k_b_ is the Boltzmann’s constant (1.38 × 10^−23^ J K^−1^), and T is the temperature (298 K). The flat band potential, V_fb_, is obtained by intersecting the initial linear slope (from flat band potential to 0.0 V vs. Ag/AgCl) with the *x*-axis, which corresponds to V_fb_ = −0.62 V (blue line) and −1.23 V (red line) for LFO and STO, respectively. 

The EIS experiment was performed using the standard three-electrode cell and tap water as the electrolyte, using Pt fishnet as the counter electrode, and Ag/AgCl as the reference electrode. The applied voltage was fixed at 1 V while performing a frequency sweep ranging from 0.01 Hz to 100 KHz under halogen lamp light irradiation with a power of 100 mW/cm^2^. The obtained result was modeled by the equivalent Randles circuit to fit the EIS Nyquist plots (Figure 8b), where R_S_, R_CT_, and C_dl_ correspond to the solution resistance, charge transfer resistance at the semiconductor–electrolyte interface, and capacitance of the electrochemical double layer, respectively (insert in Figure 8b).

The extracted Rs values of the LFO, STO, and LFO_0.5_/STO_0.5_ are 0.43, 0.27, and 0.18 KΩ, respectively, showing that the LFO_0.5_/STO_0.5_ has the smaller solution resistance. The fitted R_CT_ values of the samples correspond to the following order: LFO_0.5_/STO_0.5_ (13.82 KΩ) < STO (19.73 KΩ) < LFO (20.57 KΩ). Hence, the lower resistance to charge transfer is registered by the LFO_0.5_/STO_0.5_, which indicates that the coupling of STO and LFO has improved the conductivity and allowed an increased rate of charge transfer, in agreement with the high photocurrent density obtained for the LFO_0.5_/STO_0.5_.

The suitability of LFO_1−x_/STO_x_ films as photoanodes for photoelectrochemical WS was examined to determine the effects of the phase intermixing in enhancing the photocatalytic activity toward HER. In this sense, Nernst equations were utilized to evaluate the energies of the conduction band minimum (E_CB_) and the valence band maximum (E_VB_) for all the LFO_1−x_/STO_x_ samples. The equations include the Mulliken electronegativities (χ) and band gap energies, E_CB_ and E_VB_, as per the following equations:

$$E_{{CB}^{pH=0}}=-\frac{1}{2}\;E_{g\;}+\chi +E_{0}$$

$$E_{{VB}^{pH=0}}=\frac{1}{2}\;E_{g\;}+\chi +E_{0}$$

$$E_{{\left(CB-VB\right)}^{pH}}=E_{{\left(CB-VB\right)}^{pH=0}}-0.05911\times pH$$

**Figure 9:**
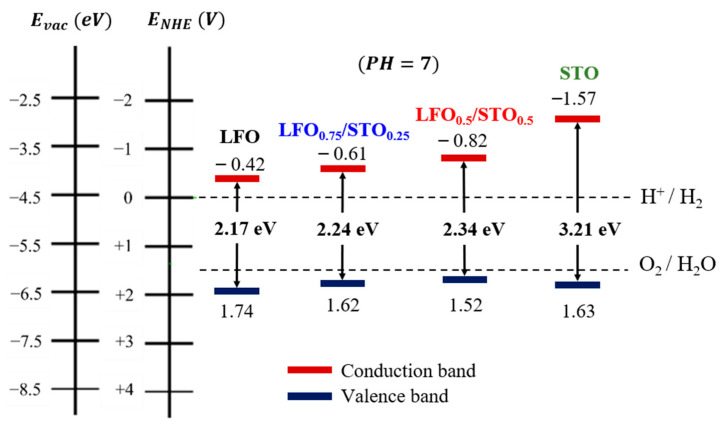
Band alignment of LFO_1−x/_STO_x_ films prepared by highly milled powders.

E_0_ is the applied potential to bring the reference redox level to the vacuum scale, taken as E_0_ = −4.5 eV. Using these equations, the band alignment with the H_2_O redox potentials (i.e., $E_{H^{+}/H_{2}}$ = 0 V and $E_{O_{2}/H_{2}O}$= 1.23 V) at pH = 7 were then determined, as depicted in Figure 9.

The pristine LFO system appears to be well aligned with the oxidation potential of water redox, which would effectively drive the OER. However, its E_CB_ minimum is not at the appropriate energy level for the HER, and further adjustment to an energy lower than $E_{H^{+}/H_{2}}$ is needed. The pure STO, on the other hand, displays good band alignment with the water redox potentials. Yet, the STO has a significant disadvantage, due to a large band gap energy of 3.28 eV, enabling only UV light absorption. It is worth noting that the LFO_1−x_/STO_x_ with intermediate composition achieves the best band alignment, coherent with high OER and HER activity, while exhibiting a relatively lower bang gap, allowing high light absorption in the visible range. This is, therefore, an additional beneficial effect of LFO and STO intermixing, especially at half content, for the increased generated current density necessary to achieve a higher hydrogen yield via the WS process. 

### 3.4. Photocatalytic Mechanism 

**Figure 10:**
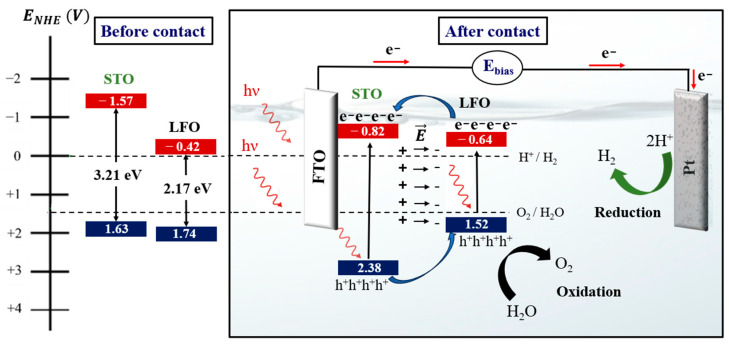
Schematic illustration of the photoelectrocatalytic mechanism taking place within the LFO_1−x_/STO_x_ nanocomposite. Light blue arrows show the injections of electrons and holes between the the LFO and STO bands, dark blue and green arrows indicate the respective sites for the oxygen and the hydrogen evolution reactions.

Figure 10 illustrates the proposed mechanism taking place at the LFO/STO interface, the origin of which is the photoelectrocatalytic performance enhancement in the LFO_1−x_/STOx nanocomposite. It should be noted that the LFO_1−x_/STOx samples exhibit an intermixing of LFO and STO grains, each with a nanometric size of approximately 50 nm. Therefore, we have depicted the interaction between two adjacent grains of LFO and STO in this figure. 

When LFO and STO grains come into contact, the conduction and valence bands of the STO undergo a downward shift, while those of the LFO experience an upward shift. This shift is a result of both pristine samples being of n-type, as confirmed by Mott–Schottky measurements.

Our findings demonstrate the presence of a built-in electric field at the interface of the STO and LFO heterojunction. This internal electric field could be responsible for facilitating the efficient transfer of photogenerated electrons from the conduction band of the LFO to the one of the STO and the accumulation of photogenerated holes from the valence band of the STO to the one of the LFO. Consequently, the oxidation reaction occurs within the LFO grains, while the electrons flow from the STO toward the platinum electrode to facilitate the reduction reaction, ultimately leading to hydrogen generation. 

## 4. Conclusions

LFO_1−x_/STO_x_ nanocomposites were successfully synthesized using a combined approach consisting of solid-state and high-energy ball milling. The resulting compounds were subsequently deposited by spray coating on an FTO substrate. The good crystallinity and purity, as well as the downsizing, were demonstrated using structural, vibrational, and analytical electron microscopy. Our findings evidenced that the intermixing between LFO and STO at 50% content each enabled a high photocurrent density yield reaching up to 11 µA cm^−2^ at 1.23 V versus RHE. Furthermore, the LFO_0.5_/STO_0.5_ yielded an applied bias photo-to-current efficiency of 4.1 × 10^−3^% at 0.76 V vs. the RHE under standard halogen lamp illumination. These encouraging results toward the development of high-yield photoanodes for hydrogen generation via a water-splitting process are ascribed to several factors, such as improved visible light absorption, high electron–hole separation, and the presence of a built-in electric field at the interface of the STO and LFO heterojunction.

## Data Availability

All data are available upon request to the corresponding author.

## Supplementary Materials

The following supporting information can be downloaded at: https://www.mdpi.com/article/10.3390/nano13212863/s1, Figure S1: Optical properties of LFO_1−x_/STO_x_ films: (a) transmittance, (b) reflectance; Figure S2: Photoelectrochemical measurements: (a) photocurrent density versus potential, (b) current density under illumination versus potential, (c) current density in the dark versus potential; Figure S3: Typical EDS spectrum recorded in STO_0.5_LF_0.5_ sample; Table S1: Element content quantification measured by EDS-SEM of all samples studies. Refs. [33,39,50,51,52] are cited in Supplementary Materials.
