# Supplementary material for: Strong Intermixing Effects of LFO1−x/STOx toward the Development of Efficient Photoanodes for Photoelectrocatalytic Applications
Source: Nanomaterials (Basel). 2023 Oct 29;13(21):2863. doi: 10.3390/nano13212863 (PMC10649736; doi:10.3390/nano13212863)
Supplement: Supplementary file 1 [file nanomaterials-13-02863-s001.zip › nanomaterials-2666910-supplementary.pdf]

Strong Intermixing Effects of LFO_{1-x}/STO_x Toward the Development of Efficient Photoanodes for Photoelectrocatalytic Applications

**Yassine Nassereddine [1], Manal Benyoussef [1], Nitul S. Rajput [2], Sébastien Saitzek [3], Mimoun El Marssi [1]
and Mustapha Jouiad [1,*]**

[1] Laboratory of Physics of Condensed Matter, University of Picardie Jules Verne, Scientific Pole,
33 Rue Saint-Leu, CEDEX 1, 80039 Amiens, France; yassine.nassereddine@u-picardie.fr (Y.N.); manal.benyoussef@u-picardie.fr (M.B.); mimoun.elmarssi@u-picardie.fr (M.E.M.)

[2] Advanced Materials Research Center, Technology Innovation Institute, Abu Dhabi P.O. Box 9639,
United Arab Emirates; nitul.rajput@tii.ae

[3] Catalyse et Chimie du Solide (UCCS), University of Artois, CNRS, Centrale Lille, ENSCL, UMR 8181, 62300 Lens, France; sebastien.saitzek@artois-univ.fr

* Correspondence: mustapha.jouiad@u-picardie.fr

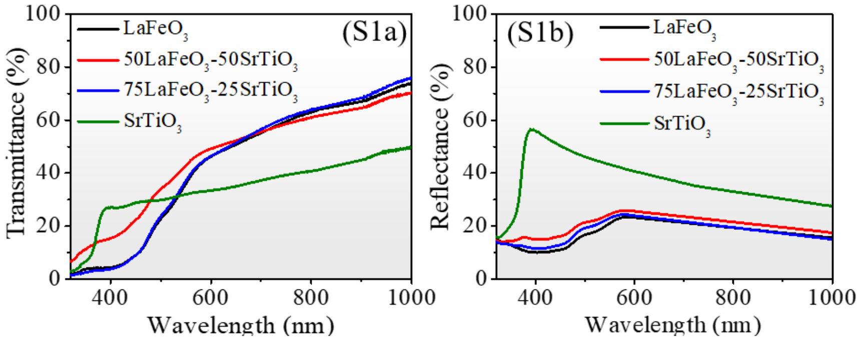

Figure S1: Optical properties of LFO_{1-x}/STO_x films: (a) transmittance, (b) reflectance

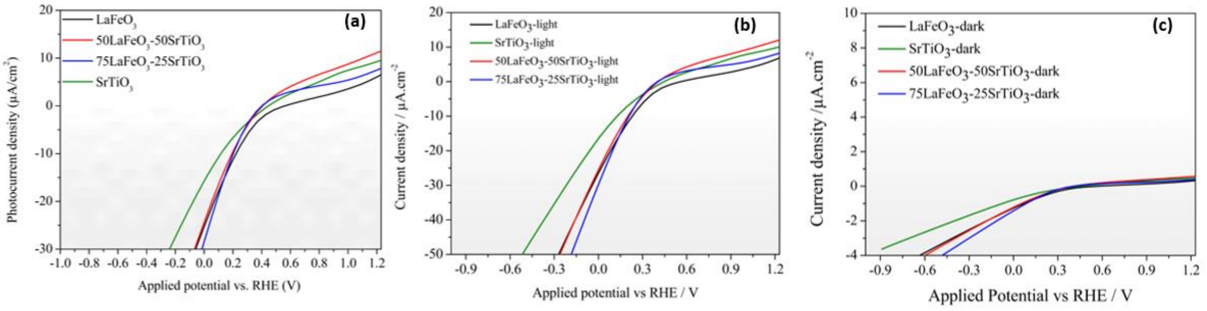

Figure S2: Photoelectrochemical measurements: (a) photocurrent density versus potential, (b) current density under illumination versus potential, (c) current density in the dark versus potential

As can be seen in Figure 7a (e.g. the manuscript) and Figure S2, negative photocurrent values were measured for voltages 0-0.2 V vs RHE for $LaFeO_3$-based compounds. Our results are similar to those reported previously [1,2]. These authors attributed this phenomenon to the co-existence of both photoanodic and photocathodic behavior in the antiferroic $LaFeO_3$-based compounds and in the multiferroic $BiFeO_3$-based compounds [3]. It has be noted that this behavior is not related to photocorrosion, generally appearing at 0.6 V vs RHE and leading to a drop in photocurrent intensity as

observed in both steady state photocurrent and transient current e.g. figure 7 (b) and (c) measured at fixed 0.6 V vs RHE for $LaFeO_3$-based compounds. This was confirmed by the unchanged measured photocurrent for $SrTiO_3$ compound (green curve in figure 7 (b) and (c)). Similar behavior was also detected in our previous works on $BiFeO_3$-based compounds [4,5].

Table S1: Element content quantification measured by EDS-SEM of all samples studies.

**Sample/elements**	**O**	**Ti**	**Sr**	**La**	**Fe**
**STO**	68.18	15.94	15.88	----	----
**LFO**	53.26	----	----	24.32	22.42
**$STO_{0.25}LFO_{0.75}$**	57.78	5.50	5.80	15.75	15.17
**$STO_{0.5}LFO_{0.5}$**	66.68	8.63	9.46	7.59	7.64

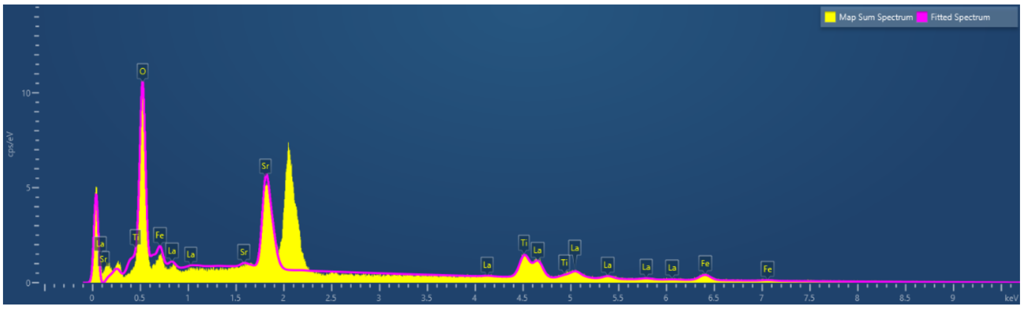

Figure S3: Typical EDS spectrum recorded in $STO_{0.5}$ $LF_{0.5}$ sample.

References

[1] F. Andrei, V. Ion, R. Bîrjega, M. Dinescu, N. Enea, D. Pantelica, M.D. Mihai, V.-A Maraloiu, V.S. Teodorescu, I.-C Marcu, et al. Thickness-Dependent Photoelectrochemical Water Splitting Properties of Self-Assembled Nanostructured LaFeO3 Perovskite Thin Films. Nanomaterials 2021, 11, 1371, doi.org/10.3390/nano11061371

[2] V. Celorrio, K. Bradley, O.J. Weber, S.R. Hall, and D.J. Fermín, "Photoelectrochemical Properties of LaFeO3 Nanoparticles," CHEMELECTROCHEM, Vol. 1, 1667, doi.org/10.1002/celc.201402192

[3] X. Yan, R. Pu, R. Xie, B. Zhang, Y. Shi, W. Liu, G. Ma, and N. Yang, "Design and fabrication of Bi2O3/BiFeO3 heterojunction film with improved photoelectrochemical performance,"

Applied Surface Science, Vol. 552, 2021, 149442, doi.org/10.1016/j.apsusc.2021.149442.

[4] M. Benyoussef, S. Saitzek, N. S. Rajput, M. Courty, M. El Marssi, and M. Jouiad, "Experimental and Theoretical Investigations of Low-Dimensional BiFeO3 System for Photocatalytic Applications," Catalysts, vol. 12, 2022, doi: 10.3390/catal12020215

[5] M. Benyoussef, S. Saitzek, N. S. Rajput, M. El Marssi, and M. Jouiad, "Effect of Sr and Ti substitutions on optical and photocatalytic properties of Bi1-xSrxFe1-xTixO3 nanomaterials," Nanoscale Adv, vol. 5, pp. 869–878, 2023, doi: 10.1039/D2NA00755J.
